# Real‐world outcomes of triplet therapy with gilteritinib, azacitidine and venetoclax in FMS‐like tyrosine kinase 3 (FLT3)‐mutated relapsed/refractory acute myeloid leukaemia: A UK single‐centre experience

**DOI:** 10.1111/bjh.70676

**Published:** 2026-07-19

**Authors:** Wei Yee Chan, Hannah Al‐Yousuf, Ho Pui Jeff Lam, Sarah Leong, Jiexin Zheng, Richard Burt, Andrew J. Wilson, Ke Xu, Kate Stringaris, Samah Alimam, Kavita Raj, Rajeev Gupta, Panagiotis Kottaridis, Anish Tailor, Rebecca Burgoyne, Marc R. Mansour, Asim Khwaja, Rob Sellar, Elspeth M. Payne, Jenny O'Nions

**Affiliations:** ^1^ University College London Hospitals, NHS Foundation Trust London UK; ^2^ University College London London UK; ^3^ GOS Institute of Child Health, UCL London UK

**Keywords:** AML, FLT3, gilteritinib, real‐world, venetoclax

## Abstract

In FMS‐like tyrosine kinase 3 (*FLT3*)‐mutated relapsed/refractory acute myeloid leukaemia, gilteritinib, azacitidine and venetoclax is an effective salvage option with an ORR of 100% (mCRc [MLFS + CRc] 93%). Sixty‐two per cent of patients treated with intention‐to‐transplant proceeded to allogeneic haematopoietic stem cell transplant, of whom 50% remain in long‐term remission (median follow‐up 43 months).

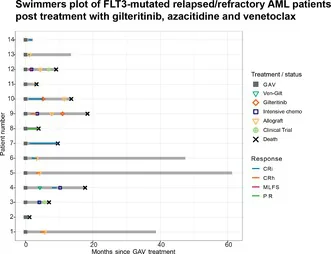

To the Editor,

Venetoclax (VEN), with the hypomethylating agent (HMA), azacitidine (AZA) is the established non‐intensive treatment for newly diagnosed (ND) acute myeloid leukaemia (AML).^1^ FMS‐like tyrosine kinase 3 mutations (FLT3mt) are present in 20%–35% of AML and associated with inferior outcomes with HMA–VEN, particularly internal tandem duplications (*FLT3*‐ITD), which confer worse prognosis in risk stratification systems such as ELN (European LeukemiaNet) 2024^2^ and PRISM.^3^ The FLT3‐inhibitor (FLT3i) gilteritinib was licensed for relapsed/refractory (R/R) FLT3mt AML following the ADMIRAL trial demonstrating improved median overall survival (mOS) of 9.3 months (m) compared to salvage chemotherapy (5.6 m).^4^ However, long‐term remission requires allogeneic haematopoietic stem cell transplant (alloHSCT) and lesser benefit from gilteritinib over salvage chemotherapy has been reported in real‐world use (mOS 7 vs. 9.6 m).^5^ AZA–VEN has demonstrated efficacy in R/R AML, with mOS of ~5–14 m, ~60% at 2 years (y) post‐alloHSCT.^6^, ^7^ Combining gilteritinib with VEN‐based regimens results in higher response rates compared to HMA–VEN in ND FLT3mt AML.^8^, ^9^ Single‐ and multicentre single‐arm studies have reported impressive CRc (Composite Complete Remission) rates with gilteritinib–AZA–VEN (GAV) of ~90%,^8^ ~80% from real‐world experience,^10^ with larger randomised studies underway.^8^, ^11^, ^12^ Data in R/R FLT3mt AML are limited to phase I/II and real‐world studies from United States and China with small patient numbers^10^, ^13^; hence, its effectiveness as a salvage regimen and as a bridge to alloHSCT in this setting remains to be fully understood. We describe the first UK and European cohort of real‐world GAV experience in R/R patients and compare this to current literature.

Fourteen consecutive patients with FLT3mt R/R AML were treated with GAV at our centre between January 2021 and December 2025, median follow up 11 m (1–61 m) (Supporting Information—Supplementary methods). Median age was 47.5 y (20–78 y) (Table 1). Twelve (86%) had *FLT3*‐ITD at diagnosis, two *FLT3*‐tyrosine kinase domain (TKD), and one had both. Variant allele frequency (VAF) was known in 11 cases, with median VAF 34% (11%–47%). Six patients (43%) had adverse and 8 (57%) intermediate risk disease by ELN2022 criteria; 12 (86%) had intermediate and 2 (14%) favourable risk disease by ELN2024; 10 (71%) had low, 3 (21%) moderate, 1 (7%) high risk by PRISM^3^ (Figure S1). Most patients (13/14, 93%) received intensive induction treatment (Table 1; Table S1); 11 (79%) were primary refractory (defined in Supporting Information—Supplementary methods)^14^ and 3 (21%) had relapsed disease. Median prior lines of treatment was one, range 1–3 (Table 1; Table S1); eight patients (57%) had prior exposure to midostaurin, four (29%) to gilteritinib monotherapy, four (29%) had prior AZA–VEN and one (7%) had relapsed post‐alloHSCT.

**TABLE 1:** Baseline characteristics in GAV and AZA–VEN cohorts.

Baseline characteristics	GAV triplet cohort (*n* = 14)	AZA–VEN cohort (*n* = 8)
Age, median years [range]	47.5 [20–78]	69.5 [31–77]
Male (%)	8 (57)	5 (62.5)
Disease status (%)		
Primary refractory	11 (79)	1 (12.5)
Relapsed	3 (21)	7 (87.5)
Diagnostic *FLT3* mutation type (%)		
ITD	11 (79)	5 (62.5)
TKD	2 (14)	2 (25)
ITD and TKD	1 (7)	1 (12.5)
Co‐mutations at diagnosis (%)		
*NPM1*	4 (39)	3 (37.5)
*WT1*	5 (36)	0
*NUP98* *(::NSD1*/*::ZFX1)*	3 (21)	0
*TET2*	3 (21)	0
*DNMT3A*	2 (14)	3 (37.5)
*RUNX1/RUNX1T1*	1 (7)	0
*ASXL1*	1 (7)	0
*DEK‐CAN*	1 (7)	0
*NRAS*	1 (7)	1 (12.5)
*KMT2A*	1 (7)	0
*IDH1/2*	1 (7)	3 (37.5)
*SRSF2*	1 (7)	0
*U2AF1*	0	1 (12.5)
*CSF3R*	0	1 (12.5)
*BCOR1*	0	1 (12.5)
*CKIT*	0	1 (12.5)
*CB1*	0	1 (12.5)
*EZH2*	0	1 (12.5)
ELN criteria at diagnosis 2017 (%)		
Favourable	0 (0)	1 (12.5)
Intermediate	6 (43)	4 (50)
Adverse	8 (57)	3 (37.5)
ELN criteria at diagnosis 2022 (%)		
Favourable	0 (0)	1 (12.5)
Intermediate	9 (64)	4 (50)
Adverse	5 (36)	3 (37.5)
ELN2024 less intensive (%)		
Favourable	2 (14)	1 (12.5)
Intermediate	12 (86)	7 (87.5)
PRISM (%)		
Low	10 (71)	2 (25)
Intermediate	3 (21)	5 (62.5)
High	1 (7)	1 (12.5)
Diagnostic cytogenetics (%)		
Favourable	0 (0)	1 (12.5)
Intermediate	12 (92)	6 (75)
Adverse	1 (8)	1 (12.5)
Induction therapy (%)		
Intensive	13 (93)	7 (87.5)
DA	0 (0)	4 (57)
DA + midostaurin	7 (54)	2 (28.6)
DA + GO	0 (0)	1 (14.3)
DA + GO + midostaurin	1 (8)	0 (0)
FLA‐IDA	5 (39)	0 (0)
Less intensive	1 (7)	1 (12.5)
AZA–VEN	1 (100)	0 (0)
AZA + cytarabine	0 (0)	1 (100)
Previous therapy and exposures (%)		
Median previous lines of therapy [range]	1 [1–3]	1 [1–3]
Previous TKI exposure	6 (46)	5 (62.5)
Previous HMA exposure	0 (0)	1 (12.5)
Previous VEN exposure	4 (31)	0 (0)
Prior HSCT	1 (8)	2 (25)
Previous gilteritinib monotherapy exposure	3 (23)	1 (12.5)
Median time [range] after C1 GAV (*n* = 11) or AZA–VEN (*n* = 7)		
Neutrophils ≥0.5 × 10^9^/L	53 [31–69]	34 [21–49]
Platelets ≥50 × 10^9^/L	37 [0–82]	31 [12–42]
Neutrophils ≥0.5 × 10^9^/L and platelets ≥50 × 10^9^/L	53 [31–82]	35 [31–49]

Abbreviations: AZA, azacitidine; DA, daunorubicin and Ara‐C (cytarabine); FLA, fludarabine; FLT3, FMS‐like tyrosine kinase 3; GAV, gilteritinib, azacitidine and venetoclax; GO, gemtuzumab ozogamicin; HMA, hypomethylating agent; HSCT, haematopoietic stem cell transplant; IDA, idarubicin and Ara‐C (cytarabine); ITD, internal tandem duplication; LDAC, low‐dose cytarabine; TKD, tyrosine kinase domain; TKI, tyrosine kinase inhibitor; VEN, venetoclax.

Patients received at least one cycle GAV (one [57%], two [36%] and three cycles [7%]; range 1–3). The majority (10/14, 71%) received 14 days VEN (range 6–24 days), 13 (93%) received 100 mg VEN daily with posaconazole and one (7%) received 400 mg VEN without azole (Table S1). Twelve patients (86%) received 80 mg gilteritinib in combination with AZA–VEN, one (7%) received 120 mg gilteritinib and one (7%) received 120–200 mg. Eleven patients (79%) recovered neutrophils to ≥0.5 × 10^9^/L after C1, with median time to recovery of 53 days (range 31–69). Two patients did not recover neutrophils, and one died while neutropenic. Six patients (43%) received >1 cycle of GAV; one relapsed on treatment, one remained aplastic (neutrophils <0.5 × 10^9^) and four (67%) recovered neutrophils to ≥0.5 × 10^9^/L after a median 29.5 days (14–42). All patients initiated C1 as inpatients; however, 11 patients (79%) required unplanned subsequent or prolonged hospital admission for febrile neutropenia (median 4 days [0–154]).

All patients achieved at least a partial remission (PR) after GAV by ELN2022^14^ (Figure 1A; Table S1); 12 patients (86%) achieved CRc (complete remission [CR] + complete remission with partial haematological recovery [CRh] + complete remission with incomplete haematological recovery [CRi]), 13 (93%) mCRc (morphological leukaemia‐free state [MLFS] + CRc) and 1 PR (7%). Median time to best response was 42.5 days (20–169) after median one cycle of GAV (1–3 cycles); best response was after one (57%), two (29%) and three (14%) cycles. Nine patients (64%) had a molecular minimal residual disease (MRD) marker (Table S1) and one (7%) achieved an MRD‐negative remission (Figure S2). Of the remaining 5 patients, two were MRD negative based on leukaemic‐associated immunophenotype. Ultrasensitive *FLT3* MRD was not available.

**FIGURE 1:**
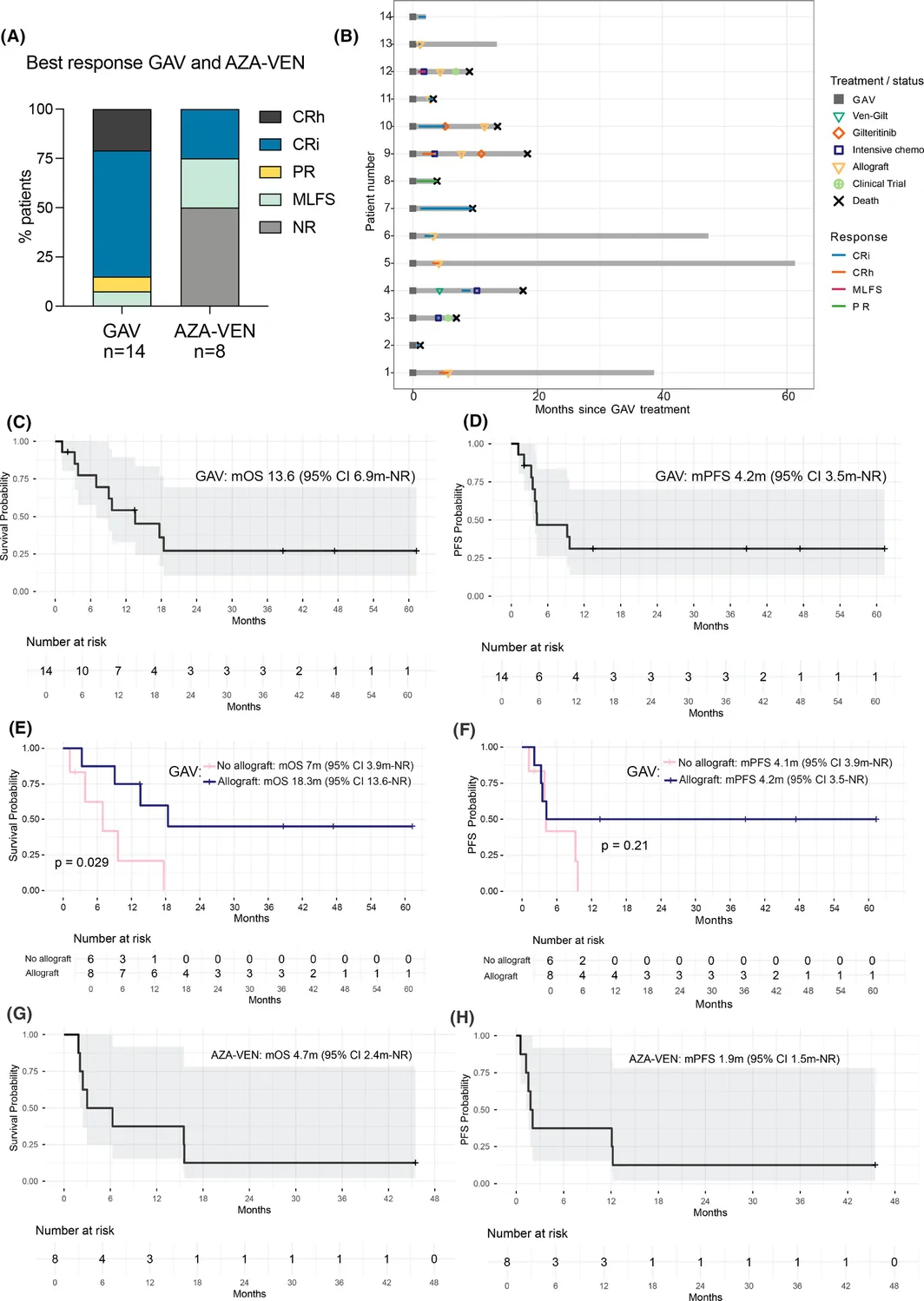
Response to GAV and AZA–VEN, overall (OS) and progression‐free survival (PFS) in GAV cohort, by allograft status and OS and PFS in AZA–VEN cohort. (A) Best response to GAV and AZA–VEN‐doublet cohort. (B) Swimmer's plot graphically demonstrating individual patient outcomes including the duration of best response and treatments received post‐GAV therapy. Intensive chemotherapy included FLA‐Ida (fludarabine, cytarabine and idarubicin) or FLA‐V‐Ida, as previous in combination with venetoclax. (C) Outcome of GAV cohort: OS from relapse episode treated with GAV, the shaded area represents the 95% confidence interval (CI). NR, not reached. (D) Outcome of GAV cohort: PFS from relapse episode treated with GAV, the shaded area represents the 95% CI. NR, not reached. (E) OS of GAV cohort by allograft. NR, not reached. (F) PFS of GAV cohort by allograft. NR, not reached. (G) Outcome of AZA–VEN cohort: OS from relapse episode treated with AZA–VEN the shaded area represents the 95% CI. (H) Outcome of AZA–VEN cohort: PFS from relapse episode treated with AZA–VEN, the shaded area represents the 95% CI. NR, not reached. GAV = gilteritinib, azacitidine and venetoclax triplet therapy. For all analyses, median OS (mOS), median PFS (mPFS) and 95% CI were calculated using the Kaplan–Meier estimator, from the time of GAV treatment. Differences between groups were assessed using the log‐rank test. AZA, azacitidine; CRh, complete remission with partial haematological recovery; CRi, complete remission with incomplete haematological recovery; GAV, gilteritinib, azacitidine and venetoclax; MLFS, morphological leukaemia‐free state; NR, no response; PR, partial remission; VEN, venetoclax. [Colour figure can be viewed at wileyonlinelibrary.com]

Thirteen patients (93%) were treated with intention to transplant (ITT) and eight (62%) underwent alloHSCT; five (63%) immediately after GAV and three (37%) required additional therapy prior to alloHSCT due to inadequate response (two received fludarabine‐idarubicin and one gilteritinib monotherapy). None requiring additional therapy post‐GAV and pre‐alloHSCT are alive at censor. At alloHSCT, seven (88%) were in CRc, and one (12%) in PR. Post‐alloHSCT, one patient (13%) died of sepsis D + 15, three (38%) relapsed, and four (50%) remain in remission (median follow‐up 43 m [range 13–61 m]). Five ITT patients (38%) did not proceed to alloHSCT after GAV, of whom two (15%) died in remission of sepsis and three (23%) were refractory to subsequent therapies. Individual patient outcomes are summarised in Figure 1B and Table S1.

The mOS for all patients was 13.6 m (95% confidence interval [CI] 7 m to not reached [NR]) and median progression‐free survival (mPFS) was 4.2 m (95% CI 3.5 m to NR). Those who underwent alloHSCT had longer mOS (18.4 vs. 7 m, *p* value = 0.029) (Figure 1C–F). Patients stratified as ELN2022 adverse risk had improved OS compared to intermediate (mOS NR vs. 9.6 m *p* = 0.046) (Figure S1A,B), with no difference seen in ELN2024 or PRISM stratified patients (Figure S1C–F), emphasising the need for refinement of prognostication systems in newer therapies and relapsed disease. No difference in outcome was observed with prior midostaurin (Figure S1G,H). Overall, five patients (36%) died of R/R AML, four (29%) of infection (one post‐alloHSCT) and five were alive in remission at data censor (Figure 1B).

We reviewed outcomes of a cohort of eight contemporaneous patients consecutively treated with AZA–VEN at our centre (Figure 1A,G,H; Table S2). All have FLT3mt AML (6 *FLT3*‐ITD, 2 TKD) but are otherwise unmatched due to the small numbers. Median age was 69.5 y (31–77 y). Seven (87.5%) had relapsed disease and one (12.5%) primary refractory after a median of one (1–3) prior line of therapy (Table 1; Table S2). Seven patients (87.5%) had received prior intensive chemotherapy, five (62.5%) prior FLT3i and three (37.5%) prior alloHSCT. By ELN2022 criteria, one (12.5%) had favourable risk disease, four (50%) intermediate and three (37.5%) adverse. ELN2024 and PRISM stratification are detailed in Table 1. The median number of AZA–VEN cycles received was 2 (1–7), containing a median 21 days of VEN (range 14–28) in C1. Time to neutrophils (≥0.5 × 10^9^/L) and time to partial haematological recovery (neutrophils ≥0.5 × 10^9^/L and platelets ≥50 × 10^9^/L) after C1 was longer with GAV compared to AZA–VEN (53 vs. 34 days, *p* = 0.006 and 53 vs. 35 days, *p* = 0.015; Table 1), in keeping with greater haematological toxicity reported for GAV.^8^, ^15^ The frequency of febrile neutropenia requiring hospital admission was comparable in both GAV and AZA–VEN cohorts (85% vs. 100%), with no apparent difference in treatment‐related mortality/deaths.

Fewer patients (*n* = 5, 62.5%) were treated with ITT, lower response rates after AZA–VEN treatment were observed compared to GAV (Figure 1A). Four patients (50%) responded and four were refractory (50%). CRc was achieved in two (25%, mCRc 50%), with median time to mCRc of 27 days (20–36 days). Fewer patients underwent alloHSCT, 12.5% (20% ITT). Comparable deaths from infection were observed after AZA–VEN cohort (12.5% vs. 15%) but higher relapse rates (75%, including one post‐alloHSCT), with one patient (12.5%) alive at data censor, after median follow‐up of 4.7 m (range 1.9–45.7 m) (Figure 1G,H).

Formal comparisons of outcomes between the GAV cohort and AZA–VEN was limited due to the very small numbers, unmatched nature of the cohorts (i.e. differences in age, mutations at baseline and proportion of relapsed and refractory patients) and the retrospective nature of the analysis. Despite these limitations we observe comparable responses with GAV in real‐world use compared to available literature. Our GAV cohort compared favourably to other reported real‐world outcomes of R/R AML treated with VEN‐based regimens (objective response rate (ORR) ~35%–50%).^6^ Variable responses with GAV have been reported; a phase I/II study of 22 patients described a CRc of 27%^8^ and two real‐world studies reported CRc of 18% and 67% in 22 and 18 patients treated with GAV, with similar mOS: 10 and 12 m.^10^, ^13^ Majority of patients in our GAV cohort were treated with ITT, with more undergoing alloHSCT compared to previous literature at 57% (62% of ITT) vs. 23%^8^ and 22%.^10^

Consistent with literature, haematological toxicity was significant with GAV. Careful patient selection, dose adjustments and management of cytopenias are key to using this regimen. We perform a bone marrow by D21 to ensure blast clearance and guide further subsequent dose reductions. Updated trial data now recommend 80 mg gilteritinib and 14 days of VEN. Ongoing studies are investigating shorter VEN duration and sequential gilteritinib dosing (NCT06696183). Our findings add to the limited published literature on this triplet therapy in the R/R setting and show GAV can be an effective bridge to alloHSCT and offer long‐term remission in selected patients with appropriate dose adjustments. These data highlight the need for larger randomised studies in R/R FLT3mt AML to better identify which patients may benefit most from novel combination therapy.

## AUTHOR CONTRIBUTIONS

**Wei Yee Chan:** Writing – original draft; writing – review and editing; formal analysis; data curation; visualization. **Hannah Al‐Yousuf:** Data curation; formal analysis; writing – original draft; writing – review and editing. **Ho Pui Jeff Lam:** Writing – review and editing; data curation. **Sarah Leong:** Writing – review and editing; data curation. **Jiexin Zheng:** Writing – review and editing. **Richard Burt:** Writing – review and editing. **Andrew J. Wilson:** Writing – review and editing. **Ke Xu:** Writing – review and editing. **Kate Stringaris:** Writing – review and editing. **Samah Alimam:** Writing – review and editing. **Kavita Raj:** Writing – review and editing. **Rajeev Gupta:** Writing – review and editing. **Panagiotis Kottaridis:** Writing ‐ review and editing. **Anish Tailor:** Writing – review and editing. **Rebecca Burgoyne:** Writing – review and editing. **Marc R. Mansour:** Writing – review and editing. **Asim Khwaja:** Writing – review and editing; data curation. **Rob Sellar:** Writing – review and editing; data curation. **Elspeth M. Payne:** Writing – review and editing; supervision. **Jenny O'Nions:** Conceptualization; writing – original draft; writing – review and editing; supervision.

## FUNDING INFORMATION

The authors declare no sources of funding.

## CONFLICT OF INTEREST STATEMENT

AJW has received honoraria from Novartis, J&J, Servier and Astellas. JO has received honoraria from Servier, Jazz, Daiichi Sankyo, Astellas, Abbvie, J&J.

## Supporting information


**Figure S1.** Outcomes by ELN2022, ELN2024, PRISM risk stratification (stratified into favourable, intermediate and adverse risk groups) and by previous midostaurin exposure in GAV cohort.
**Figure S2.** MRD measurements in the GAV cohort per 100 000 ABL copies.
**Table S1.** GAV—gilteritinib, azacitidine and venetoclax patient summaries.
**Table S2.** AZA–VEN patient summaries.

## Data Availability

Data that support the findings of this study are available upon reasonable request from the corresponding author.
