# Supplementary material for: Real‐world outcomes of triplet therapy with gilteritinib, azacitidine and venetoclax in FMS‐like tyrosine kinase 3 (FLT3)‐mutated relapsed/refractory acute myeloid leukaemia: A UK single‐centre experience
Source: Br J Haematol. 2026 Jul 19;209(3):1386–90. doi: 10.1111/bjh.70676 (PMC13570139; doi:10.1111/bjh.70676)
Supplement: Supplementary file 1 [file BJH-209-1386-s001.docx]

**Supplementary Methods**

The service evaluation was performed at University College London Hospitals NHS Foundation Trust from January 2021 and December 2025. Consecutive patients treated with Gilteritinib, Azacitidine and Venetoclax (GAV) were included in the analysis. Consecutive patients with a FLT3mt treated with AZA-VEN from December 2020 to February 2022 were included as a contemporaneous comparison cohort. Primary refractory was defined as per ELN 2022 as failing to achieve response after minimum 2 cycles of intensive chemotherapy or at predetermined landmark after less-intensive therapy. Partial haematologic recovery was defined as per ELN 2022 (neutrophils ≥0.5 x 10^9^/L and platelets ≥50 x 10^9^/L).

Descriptive analyses were reported as median with range for age and time. Unpaired medians were compared by Mann-Whitney U test. Median overall survival (mOS), median progression-free survival (mPFS) and 95% confidence interval (CI) were calculated using the Kaplan-Meier estimator, from the time of GAV or AZA-VEN treatment. Differences between groups were assessed using the log-rank test.

Ultrasensitive FLT3 MRD was not available at the time of this cohort receiving treatment. Available alternative MRD markers to FLT3 (detailed in supplementary figure 2, supplementary table 1) was measured using Qiagen Rotorgene RGQ. Assay sensitivity was reported to 1 BCR-ABL transcript per 100 000 ABL copies (1 per 10^5^).

**Supplementary Figure 1: Outcomes by ELN2022, ELN2024, PRISM risk stratification (stratified into favourable, intermediate and adverse risk groups) and by previous midostaurin exposure in GAV cohort**

**S1A:** Median overall survival (mOS) by ELN 2022 at diagnosis. **S1B:** Median progression free survival (mPFS) by ELN 2022 at diagnosis. **S1C:** mOS by ELN 2024 at diagnosis. **S1D:** mPFS by ELN 2024 at diagnosis. **S1E:** mOS by PRISM at diagnosis **S1F:** mPFS by PRISM at diagnosis **S1G:** mOS by previous midostaurin exposure. **S1H:** mPFS by previous midostaurin exposure. For all analyses, mOS, mPFS and 95% CI were calculated using the Kaplan-Meier estimator, from the time of GAV treatment. Comparisons between groups were calculated using the log-rank test. NR, not reached.

**Supplementary Figure 2: MRD measurements in the GAV cohort per 100 000 ABL copies**

Y axis is the MRD measurement per 100 000 ABL copies, with a dotted line drawn at the level of 1 per 100 000 ABL copies. X axis represents the available MRD measurement after each cycle of GAV. Each patient id corresponds to that reported in Figure 1B and row number of Supplementary Table 1. All MRD was measured using Qiagen Rotorgene RGQ. Assay sensitivity was reported to 1 BCR-ABL transcript per 100 000 ABL copies.

**Supplementary Table 1: GAV – Gilteritinib, Azacitidine and Venetoclax Patient Summaries**

| **Age, Sex** | **Diagnosis** | **ELN 2022** | **ELN 2024** | **PRISM** | **NPM1** | **FLT3 mutation type (diagnosis)**  **(VAF %)** | **Molecular MRD marker** | **Induction regimen** | **Other prior treatment** | **Previous lines** | **Gilteritinib**  **Dose** | **VEN**  **Dose (mg)** | **Best response** | **Time to best response (days)** |
| --- | --- | --- | --- | --- | --- | --- | --- | --- | --- | --- | --- | --- | --- | --- |
| 26, M | Relapsed AML | Adverse | Favourable | Low | Neg | TKD (na) | KMT2A-ELL  (MLL-ELL) | DA + Midostaurin | Fla-IDA | 2 | 80mg with VEN, 120mg after | 100 | CRh | 169 |
| 72, F | Primary refractory AML | Intermediate | Intermediate | Intermediate | Pos | ITD (11) | NPM1 | DA + Midostaurin | NA | 1 | 80mg with VEN, 120mg after | 100 | CRi | 20 |
| 62, F | Primary refractory AML | Intermediate | Intermediate | Low | Pos | ITD (47) | NPM1 | DA+ Midostaurin | Gilteritinib alone | 2 | 120mg | 100 | CRi | 117 |
| 50, M | Primary refractory AML | Intermediate | Intermediate | Low | Neg | ITD (18.1) | na | DA+GO+  Midostaurin | Gilteritinib alone | 2 | 80mg with VEN, 120mg after | 100 | CRi | 239 |
| 39, F | Primary refractory AML | Adverse | Intermediate | Intermediate | Neg | ITD (na) | na | DA+  Midostaurin | NA | 1 | 80mg with VEN, 120mg after | 100 | CRh | 95 |
| 23, F | Primary refractory AML | Adverse | Intermediate | Low | Neg | ITD (45) | DEK-CAN | Fla-IDA | NA | 1 | 80mg with VEN, 120mg after | 100 | CRi | 58 |
| 53, M | Primary refractory AML | Intermediate | Intermediate | Low | Pos | ITD (55)/TKD (na) | NPM1 | DA+  Midostaurin | Gilteritinib alone | 2 | 80mg with VEN, 120mg after | 100 | CRi | 38 |
| 52, F | Primary refractory AML | Intermediate | Intermediate | Low | Neg | ITD (30) | na | Fla-IDA | Ven-Aza, Gilteritinib alone | 3 | 200mg with VEN | 100 | PR | 20 |
| 37, M | Relapsed AML | Intermediate | Favourable | Low | Pos | TKD (na) | NPM1-MLF | Fla-IDA and allograft | NA | 1 | 80mg | 400 (No azole) | CRh | 47 |
| 45, M | Primary Refractory AML | Intermediate | Intermediate | Low | Neg | ITD (17) | NUP98::NSD1 | DA+  Midostaurin | FLA-Ida | 2 | 80mg with VEN, 120mg after | 100 | CRi | 30 |
| 53, M | Relapsed AML | Adverse | Intermediate | Intermediate | Neg | ITD (43) | na | Fla-IDA | NA | 1 | 80mg with VEN, 120mg after | 100 | CRi | 76 |
| 20, F | Primary refractory MDS EB2 | Intermediate | Intermediate | Low | Neg | ITD (30) | NUP98:  ZKF1 | DA+  Midostaurin | NA | 1 | 80mg with VEN, 120mg after | 100 | MLFS | 26 |
| 31, M | Primary refractory AML | Intermediate | Intermediate | Low | Neg | ITD (41) | NUP98:  NSD1 | Fla-IDA | NA | 1 | 80mg | 100 | CRi | 28 |
| 78, M | Primary refractory AML | Adverse | Intermediate | High | Neg | ITD (34) | na | Ven-Aza | NA | 1 | 80mg | 50 | CRi | 29 |

DA – Daunorubicin, Ara-C, FLA-IDA – Fludarabine, Ara-C and Idarubicin, GO- Gemtuzumab Ozogamicin, NA – not applicable, na – not available

**Supplementary Table 2: AZA-VEN Patient Summaries**

| **Age, Sex** | **Diagnosis** | **ELN 2022** | **ELN 2024** | **PRISM** | **NPM1** | **FLT3 mutation type (diagnosis)**  **VAF %)** | **Molecular MRD marker** | **Induction regimen** | **Other prior treatment** | **Previous lines** | **VEN dose mg** | **Best response** | **Time to best response** |
| --- | --- | --- | --- | --- | --- | --- | --- | --- | --- | --- | --- | --- | --- |
| 71  F | Refractory AML | Adverse | Intermediate | High | Neg | ITD (35) | na | Aza, Ara-C | NA | 1 | 100 | NR | NA |
| 71  M | Relapsed AML | Intermediate | Intermediate | Intermediate | Pos | ITD (0.05) | NPM1 | DA, HIDAC + midostaurin | Gilteritinib, FLA-IDA | 3 | 50 | CRi | 33 |
| 31  M | Relapsed AML | Intermediate | Favourable | Low | Neg | TKD (29.4) | na | DA | Aza + Sorafenib, Mitoxantrone + HIDAC + AlloHSCT | 3 | 100 | NR | NA |
| 49  M | Relapsed AML | Adverse | Intermediate | Intermediate | Neg | ITD (na) | na | DA + midostaurin | NA | 1 | 100 | NR | NA |
| 65  M | Refractory AML | Favourable | Intermediate | Intermediate | Neg | TKD (na) | RUNX1-RUNX1T1 | DA, HIDAC | FLA-IDA | 2 | 100 | NR | NA |
| 77  F | Relapsed AML | Intermediate | Intermediate | Intermediate | Pos | TKD (na), ITD (na) | na | DA+GO, DA, HIDAC | NA | 1 | 50 | MLFS | 21 |
| 68  M | Relapsed AML | Adverse | Intermediate | Intermediate | Neg | ITD (na) | na | DA, HIDAC + midostaurin | FLA-IDA | 2 | 100 | MLFS | 36 |
| 76  F | Relapsed AML | Intermediate | Favourable | Low | Pos | ITD (na) | NPM1 | DA + midostaurin | NA | 1 | 50 | CRi | 20 |

DA – Daunorubicin, Ara-C, HIDAC – High-dose Ara-C, FLA-IDA – Fludarabine, Ara-C and Idarubicin, GO- Gemtuzumab Ozogamicin, NA- Not Applicable, na – not available
